# The Association between Obesity and Depression among Children and the Role of Family: A Systematic Review

**DOI:** 10.3390/children9081244

**Published:** 2022-08-18

**Authors:** Aikaterini Kanellopoulou, George Antonogeorgos, Konstantinos Douros, Demosthenes B. Panagiotakos

**Affiliations:** 1 Department of Nutrition and Dietetics, School of Health Science and Education, Harokopio University, 17671 Athens, Greece; 2 Allergology and Pulmonology Unit, 3Rd Paediatric Department, National and Kapodistrian University of Athens, 12462 Athens, Greece; 3 Faculty of Health, University of Canberra, Canberra 2617, Australia

**Keywords:** childhood, depression, family role, obesity, public health

## Abstract

One of the most critical factors that affects or leads to obesity is depression. However, another point of view is that obesity leads to depression. This systematic review estimates evidence arising from observational and systematic studies concerning the association between obesity and depression in children and adolescents. Moreover, the role of the family environment is investigated in this review. A systematic literature search was performed for research conducted between 2014 and 2021 on PubMed. The basic inclusion criteria were the language, study issue and type, and age of the participants. Studies that examined non-healthy populations, or were not related, or with no access were excluded. Titles and abstracts were screened independently, and full-text manuscripts meeting inclusion criteria were extracted. Finally, twenty-seven studies were retained. Most of them highlighted a positive association between obesity and depression. However, it is not clear whether obesity leads to depression or vice versa. Our review also revealed that the role of the family in this association has not been well studied and understood, since only one study addressed the issue. The evidence from our review emphasizes major public health issues; therefore, appropriate health policies should be developed. Moreover, additional research is required to fully understand the role of the family environment in the association between depression and obesity in childhood.

## 1. Introduction

Childhood obesity (excessive fat accumulation) and depression (a mental state of a low mood and an aversion to activity) are among the most notorious public health challenges worldwide due to their high rates of prevalence, morbidity, and mortality [1,2]. Therefore, a large amount of the literature has dealt with the factors that influence the development of obesity and depression. More specifically, the prevalence of childhood obesity is 19.7% [https://www.cdc.gov/obesity/data/childhood.html (accessed on 4 August 2022)], while the prevalence of childhood depression is 4.4% [https://www.cdc.gov/childrensmentalhealth/data.html (accessed on 4 August 2022)]. Although there is a relationship between obesity and depression, their interaction is still vague. Childhood obesity is connected with an increased risk of various diseases, such as diabetes, cardiovascular diseases, stroke, certain types of cancer later in life, social problems, and depression among youth [3]. Depression may occur at any time, may lead to a variety of emotional and physical problems, and may decrease a person’s ability to function at any place [4].

Children with obesity experience numerous psycho-social problems that significantly affect their quality of life and wellbeing [5]. A systematic review conducted by Rankin et al. [6] documented that childhood overweight/obesity was negatively associated with several psychological comorbidities, with depression among them. According to the systematic review of Mühlig et al. [7], most of the cross-sectional studies supported a significant association between obesity and depression. Only three longitudinal studies reported associations between obesity and subsequent depression in female children and adolescents. The vague relationship between obesity and depression was also recognized by Small and Aplasca [8].

The literature research of Nemiary et al. [2] could not identify causality between adolescent obesity and depression; however, several indirect pathways and experiences, such as stressful life events, victimization by peers, and teasing about weight status may be contributing factors to the development of depression in adolescents. A few studies have reported that girls with obesity have a greater risk of developing depression and anxiety (social anxiety) with weight gain [9,10]. Sagar and Gupta [5] provided an evidence-based comprehensive overview of the psychological and psychiatric factors (such as depression, anxiety, eating disorders, stress, body shape concerns, and low self-esteem) related to childhood obesity that can be further used in assessing and managing this epidemic. Most of these factors can be determined by the family environment or special conditions [11,12]. Several physiological, cognitive, behavioral, and social mechanisms may be responsible for the pathway between obesity and depression, and vice versa. For more information, the interested reader is referred to the study of Markowitz et al. [13].

A family is a structure of persons, in which a child from birth receives various stimuli concerning various aspects of his or her life. Within the environment of a family, a child shapes his or her character and personality, develops his or her sociability, adopts positive or negative eating behaviors, general beliefs, several reactions to issues in his or her daily life, etc. For the purposes of this paper, the notion of family environment was considered identical to family structure, under the general meaning of the term.

From the foregoing evidence, it is not clear whether depression leads to obesity or obesity leads to depression in combination with a family context. Consequently, the aim of the present study sought (a) to systematically review the evidence of the association between childhood obesity and depression and (b) to examine the role that family environment (and in particular family structure) plays in the above association.

## 2. Materials and Methods

### 2.1. Search Strategy

In order to review the current literature regarding the association between obesity and depression in children, a systematic literature search was conducted on PubMed. Searches covered the period from January 2014 to February 2021, given that a similar review paper covered the topic until August 2014 [14,15]. The search query, including the relevant keywords and the number of papers retrieved by each keyword combination, is presented in Table 1. This systematic review followed the procedures suggested by STROBE [16] and PRISMA reporting guidelines [17].

### 2.2. Inclusion Criteria

In this review, eligible to be included were studies that (1) were written in the English language, (2) focused on the association between obesity and depression (regardless of the direction of the association), (3) focused on the role of family in the above-mentioned association, (4) included children and adolescents from early childhood to eighteen years of age, (5) had designs that were cross-sectional, longitudinal, case-control, and that were systematic reviews or meta-analyses.

### 2.3. Exclusion Criteria

**Figure 1:**
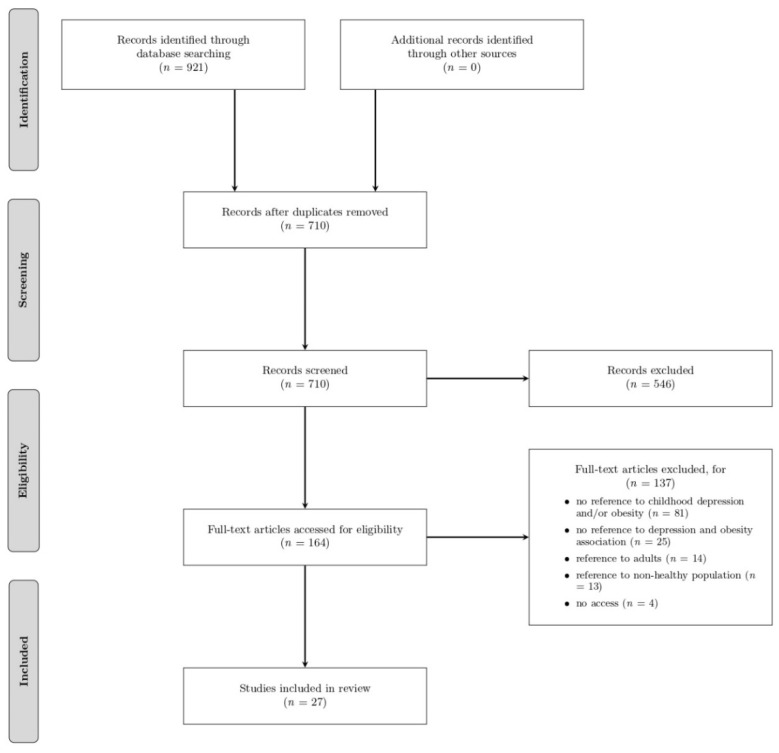
PRISMA diagram of search strategy.

Exclusion criteria were used for studies that (1) did not refer to childhood depression and/or obesity, (2) did not examine the association between obesity and depression in childhood (regardless of the direction of the association), (3) investigated the association between obesity and depression (regardless of the direction of the association) in unhealthy populations with other mental and/or physical health problems, or diseases or any other type of health disorder, (4) included participants (over eighteen years-old), (5) could not be accessed (only the abstract was available). The excluded studies in the final stage are presented in Figure 1.

### 2.4. Data Extraction

Two researchers (A.K. and G.A.) independently selected potentially relevant articles, reviewed the main documents, and extracted the relevant information, such as the year that every study was published, authors’ names, the sample and country details, and the type of study and main findings. In case of a disagreement, the other two authors (K.D. and D.B.P.) made a decision.

### 2.5. Risk of Bias Assessment

Given that there is no existing tool for studies of attitudes and practices, we assessed the risk of bias of the included studies through five items: the representativeness of the sample, adequacy of the response rate, missing data within completed questionnaires, conduct of pilot testing, and use of validated instruments [https://www.evidencepartners.com/resources/methodological-resources/risk-of-bias-cross-sectional-surveys-of-attitudes-and-practices (accessed on 11 August 2022)]. The risk of bias was characterized as “low”, “high”, or “unclear”. The third category indicated either lack of information or uncertainty over the potential for bias. For the systematic reviews and meta-analyses, the risk was assessed according to whether these had been carried out following accepted guidelines. Two researchers (A.K. and G.A.) independently assessed the risk of bias.

## 3. Results

In total, 710 unique items were retrieved. From those, 164 papers passed the first screening. The result of the reviewing process was that 27 out of the 164 items met the inclusion criteria (described in more detail in the following subsection and presented in Table 2 and Table 3), and thus were included in this systematic review.

### 3.1. Does Depression Lead to Obesity?

#### 3.1.1. Case-Control Studies

Esposito et al. [18] carried out a case-control study to evaluate the relationship between psychological problems and obesity among a sample of 148 children (8 to 12 years of age). The results of this study showed a significantly higher level of depressive and anxious symptomatology among the group with obesity in comparison with the control group. The case-control study of Cerniglia et al. [19] concluded that normal-weight children had lower depressive symptoms compared with overweight youths. Moreover, overweight females showed lower depressive scores than overweight males at 2 years of age, but they surpassed boys before 5 years of age.

#### 3.1.2. Cross-Sectional Studies

A study by Chung et al. [20] conducted on 302 children (156 children who were overweight or obese and 145 healthy-weight children) from Taipei, Taiwan revealed no significant difference in depression between the healthy-weight and overweight or obese children. Similarly, the recent network analysis of Byrne et al. [21] on 248 children and adolescents concluded that depressive symptoms and obesity were not associated. On the contrary, a study by Lynch et al. [22], on a convenience sample of 147 children 10 to 12 years-old, revealed that depressive symptoms explained a significant amount of the variance in the body mass index (BMI) and central adiposity, after adjusting for gender, race/ethnicity, puberty, and socioeconomic status.

#### 3.1.3. Longitudinal Studies

The longitudinal study of Wickrama et al. [23] showed that adolescents with more depressive symptoms and those with more genetic risk alleles, in general, had a higher BMI compared with adolescents with fewer depressive symptoms and risk alleles. Similarly, the population-based study of Schwartz et al. [24] showed that children who had experienced at least one depressive event had a higher average BMI than children without such an experience; the older the children, the stronger the association. The study of Olive et al. [25] on a sample of 791 healthy Australian children (7–8 years of age) revealed that both boys and girls with higher depressive symptoms had a higher percentage of body fat, and longitudinally, boys whose depressive symptoms increased became fatter. In the study of Nagata et al. [26], conducted on 14,322 U.S. adolescents, depressive symptoms were associated with increased odds of unhealthy weight control behaviors in females; no association was found in boys. Finally, the study of Pine et al. [27] on 231 preschoolers in St. Louis, MI, USA reported a significant positive association between preschool depressive symptoms and adolescent BMI.

#### 3.1.4. Systematic Reviews and Meta-Analyses

A recent systematic review and meta-analysis of 28 observational studies [28], from which three were prospective cohorts and 25 were cross-sectional studies, revealed no association between overweight/obesity and a risk of depression and anxiety. More specifically, the pooled risk estimate for overweight was equal to 1.00 (95% CI: 0.92–1.08, *p* = 0.97), while the pooled risk estimate for obesity was equal to 1.08 (95% CI: 0.97–1.21, *p* = 0.17).

### 3.2. Does Obesity Lead to Depression?

#### 3.2.1. Case-Control Studies

A case-control study by Topçu et al. [29] revealed that obesity in children was associated with a significantly higher rate of depression and anxiety, and lower self-esteem scores. There were significant differences among obese and control groups in terms of the total score of the children’s depression inventory (CDI) [12 (4–39)] versus [8 (3–19)]; *p* < 0.001. According to the results of the case-control “ANOBAS” study, conducted on 100 preadolescents aged 8 to 12 years from Madrid, Spain, preadolescents with obesity reported higher levels of depression [30]. A recent study conducted on 12,507 Swedish children aged 6–17 years highlighted obesity as a significant risk factor for anxiety and depression [31]. Girls with obesity had a 43% higher risk of anxiety and depression compared with girls in the general population. A similar result held for boys (adjusted HR, 1.33).

#### 3.2.2. Cross-Sectional Studies

The study of Sepulveda et al. [32] on 170 children aged 8 to 12 years from different health centers in Madrid, Spain revealed that 5% of the sample was diagnosed with major depression. According to Yang et al. [33], childhood obesity can cause depression and reduce children’s quality of life because of their distorted body perception. The study of Blanco et al. [34] on 50 Spanish preadolescents with obesity matched with non-overweight children, showing that children with obesity reported higher depression. Sepúlveda et al. [35] identified a positive association between weight status and depression (measured through CDI). However, a recent retrospective study on 445 Taiwanese children aged 13–15 years [36] showed no difference in depression between normal-weight and children with overweight/obesity (4.85 ± 3.53 vs. 4.81 ± 3.96).

#### 3.2.3. Longitudinal Studies

Lim et al. [37] classified obesity among the meaningful variables of depressive symptoms from childhood to adolescence. Other risk factors were gender, self-esteem, family structure, family conflict, peer attachment, discussion of personal issues, academic performance, and school adaptability. Gibson et al. [38] showed significant main effects of baseline weight status on girls’ depressive symptoms, after adjusting for child age, maternal BMI, and family income. Girls with overweight or obesity reported greater impairment in depression as well as in other areas of psychological difficulties than normal-weight girls. No such association was found for boys.

#### 3.2.4. Systematic Reviews and Meta-Analyses

A high depression prevalence in children with overweight or obesity was identified in a systematic review of Australian children and adolescents [39]. The meta-analysis of Quek et al. [40], including 18 studies and 51,272 participants, demonstrated a positive association between childhood and adolescent obesity and depression (pooled odds ratio = 1.34, 95% confidence interval [CI]: 1.1–1.64, *p* = 0.005) and more severe depressive symptoms (SMD = 0.23, 95% CI: 0.025–0.44, *p* = 0.028) in the obese groups. Overweight subjects were not more likely to have either depression (pooled odds ratio = 1.16, 95% CI: 0.93–1.44, *p* = 0.19) or depressive symptoms (SMD = 0, 95% CI: −0.101 to 0.102, *p* = 0.997). An umbrella review of evidence from meta-analyses and Mendelian randomization studies conducted by Köhler et al. [41] identified obesity and metabolic abnormalities as risk factors for depression. A recent systematic review and meta-analysis of cross-sectional and longitudinal observational studies conducted by Sutaria et al. [42] revealed that girls with overweight or obesity have significantly increased odds of concurrent and future depression compared with girls without obesity. More specifically, twenty-two studies representing 143,603 children were included in the meta-analysis. Almost one out of ten children with obesity were found with depression. Compared with normal-weight children, children with obesity were 32% more likely to have depression (OR 1.32, 95% CI: 1.17 to 1.50). Girls with obesity were 44% more likely to have depression (OR 1.44, 95% CI 1.20 to 1.72) than normal-weight girls. However, there was no association between overweight children and depression (OR 1.04, 95% CI 0.95 to 1.14) or among males with obesity or overweight and depression (OR 1.14, 95% CI 0.93 to 1.41% and 1.08, 95% CI 0.85 to 1.37, respectively). Separately taking into account cross-sectional and longitudinal studies revealed an association of childhood obesity with both concurrent (OR 1.26, 95% CI 1.09 to 1.45) and prospective odds (OR 1.51, 95% CI 1.21 to 1.88) of depression. On the other hand, a recent literature review [43] showed that psychological conditions, such as low self-esteem, depression, and eating disorders can result from excess weight in childhood.

### 3.3. Does the Family Environment Affect the Association between Obesity and Depression?

Only one of the retrieved studies concerned the role of the family environment in the association between obesity and depression. More specifically, Aparicio et al. [44] stated that parents influence the development of emotion regulation, a disorder of which is depression, and obesity in children can also be affected by parents, as role models and through their parenting style and parental feeding practice. Effective emotion regulation skills can decrease obesity-related unhealthy behavior and enhance protective factors, which boost mental and physical health. As a result, effective emotion regulation can contribute to the prevention and treatment of childhood obesity.

### 3.4. Risk of Bias Assessment Results

**Figure 2:**
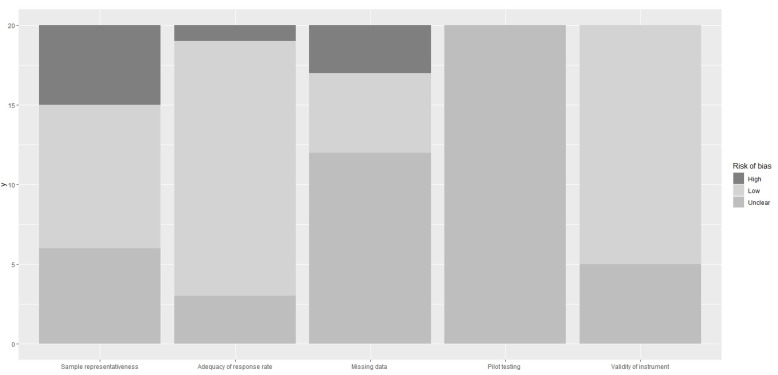
Risk of bias assessment.

The bias risk assessment (Figure 2) showed that the quality of the individual studies included in this review was high. The majority of the studies used representative samples and validated research tools, while the response rate was adequate. All the studies had unclear information about pilot testing, while more than half of them had unclear information about the existence of missing values. All systematic reviews and meta-analyses had been carried out following accepted guidelines. However, the risk of bias assessment should be considered with great caution, as due to the type of studies (epidemiological studies, systematic reviews, and meta-analyses), their authors did not make a clear or even any reference to sources of bias.

## 4. Discussion

In the present review, we attempted to shed light on or even unravel the vague relationship between obesity and depression in childhood, using the latest research findings. According to the summarized published evidence, we conclude that the relationship between them is unclear. First, there is no systematic association between depression and obesity in childhood, and second, when they are associated, it is not obvious whether depression leads to obesity or vice versa. To this end, we reviewed all case-control, cross-sectional, and longitudinal studies, as well as systematic reviews and meta-analyses published in the last seven years. In order to better study the relationship between obesity and depression, we based our research on two axes, depending on which, obesity or depression leads to the other. Moreover, we tried to investigate the role of family on the above association, something that has not been adequately studied.

### 4.1. Does Depression Lead to Obesity?

Two case-control studies agreed that children with overweight/obesity were more depressed than children with normal weight [18,19]. Two out of three cross-sectional declared no association between depression and obesity [20,21], while one study concluded that depressive symptoms explained a significant amount of the variance in BMI [22]. All six longitudinal studies identified a positive association between depressive symptoms and BMI [23,24,25,26,27]. Only one recent meta-analysis [28] revealed that there is no association between overweight or obesity and the risk of depression. However, in this paper, social, health, and lifestyle factors were not taken into account. Furthermore, there was heterogeneity in the assessment of depression and obesity in the included studies.

### 4.2. Does Obesity Lead to Depression?

Three case-control studies included in this review agreed that there were significant differences in depression between the obese and control groups [29,30,31]. Four out of five cross-sectional studies identified a positive association between obesity and depression [32,33,34,35], while the fifth one reported no significant difference in depression between children with overweight/obesity and children with normal weight [36]. Two longitudinal studies concluded that obesity was a risk factor for depression [37,38]; in the latter study, the result held only for females. All five systematic reviews and meta-analyses agreed that excess weight constituted a risk factor for depression [39,40,41,42,43]. In the meta-analysis of Sutaria et al. [42], the positive association between obesity and depression held only for girls.

In summary, 11 studies dealt with whether depression leads to obesity, while 15 studies dealt with the reverse path. Eight out of the eleven studies and 14 out of the 15 studies reported a positive association between obesity and depression. This contrasted with the literature review of Fabricatore and Wadden [45] concerning the association of obesity with psychopathology, and specifically with regard to depression; this review reported no systematic association between childhood obesity and psychopathology. However, a specific group of persons with obesity, such as girls and persons with extreme obesity, had a higher risk to develop a psychiatric disorder or emotional disturbance. Similarly, another systematic review of 17 studies reported no association between obesity and depression in children and adolescents [46].

According to the findings of our review, it would be unwarranted to claim, based on the number of studies, that obesity leads to depression. This is a question that remains unanswered and more studies are needed on it. However, regardless of the direction of the relationship that will be examined, the most reliable sources of information are prospective studies, as all other types of studies cannot determine causality. The association between obesity and depression is not a straightforward matter. Future studies should also investigate the biological pathways that may mechanistically explain the association between obesity and depression. The review of Milaneschi et al. [47] focused on biological mechanisms that might explain this association at different levels, including genes and peripheral endocrine, immuno-inflammatory processes, and metabolic mechanisms in the brain.

Our review also addressed the role that family environment may play in the association between obesity and depression. However, only one study was retrieved. Parents serve as role models and thus, they influence the development of emotion regulation, disorders of which are depression as well as obesity in children. Parenting style and parental feeding practice may affect the association between obesity and depression [44].

The reader should evaluate the results of this literature review with particular care as the evaluation of depression has been differently conducted in different studies; this is the most important limitation of the present review. However, the main research tool used in most studies was the children’s depression inventory (CDI), which was realized in its final version by Kovacs [48]. Moreover, there was heterogeneity among the studies regarding different populations, different sample sizes, differences in statistical analyses, differences in confounding factors, and the country in which each study was conducted. In addition, in this paper, we did not delve into the subject from a medical perspective. Another limitation of the review was that the search was conducted only in one database, i.e., PubMed. However, PubMed is the most widely used and complete database that comprises, at the moment this review paper was written, more than 34 million citations for biomedical literature. Despite the limitations, our investigation compiled the most recent literature and showed the opportunities for further research; the role of the family in the bidirectional relationship between obesity and depression needs more attention and study.

## 5. Conclusions

Our systematic review showed that an association between obesity and depression exists, regardless of the direction examined. Although from the number of studies, it seems that there is a tendency of researchers to consider and therefore study whether obesity leads to depression, an overall conclusion is precarious. Childhood obesity and childhood depression, both individually and collectively, are significant public health problems that may compound children’s health issues [49] or may adversely affect individuals in their adult lives and consequently society as a whole [50]. Therefore, policymakers should pay special attention to it, developing appropriate policies, actions, and/or recommendations that will address these two scourges. The study of biological pathways that lead to the unidirectional or bidirectional association between obesity and depression may assist to this end. Moreover, there is a knowledge gap in the association between childhood obesity and depression, that our review highlighted, related to whether and how the family environment affects this association.

## Figures and Tables

**Table 1:** Number of records retrieved per keyword combination.

Keyword Combinations	No of Records
(childhood obesity) AND (childhood depression) AND (family role)	27
(childhood obesity) AND (childhood depression) AND (family structure)	12
(childhood obesity) AND (childhood depression)	362
(childhood obesity) AND (childhood mental health) AND (family role)	38
(childhood obesity) AND (childhood mental health) AND (family structure)	15
(childhood obesity) AND (childhood mental health)	467
Total	921

**Table 2:** Characteristics of studies that evaluated the association between depression and childhood obesity.

Author (Year)	Sample and Country	Study Type	Main Findings
Esposito et al. (2014) [18]	148 children (8 to 12 years of age)	Case-control study	Significantly higher level of depressive and anxious symptomatology among the group with obesity in comparison to the control group
Cerniglia et al. (2018) [19]	180 children (2 to 8 years of age)	Case-control study	Normal-weight children had lower depressive symptoms compared with overweight youths. Overweight females showed lower depressive scores than overweight males at 2 years of age.
Chung et al. (2015) [20]	302 children from Taipei, Taiwan	Cross-sectional study	No significant difference in depression between the healthy-weight and overweight children or with obesity.
Byrne et al. (2021) [21]	248 children (8 to 17 years-old)	Cross-sectional study	No association between depressive symptoms and obesity
Lynch et al. (2019) [22]	147 children (10 to 12 years-old)	Cross-sectional study	Depressive symptoms explained a significant amount of the variance in the body mass index (BMI) and central adiposity when gender, race/ethnicity, puberty, and socioeconomic status were controlled
Wickrama et al. (2014) [23]	12,424 adolescents (12 to 19 years of age)	Longitudinal study	Adolescents with more depressive symptoms and adolescents with more genetic risk alleles had a higher BMI compared with adolescents with fewer depressive symptoms and risk alleles.
Schwartz et al. (2016) [24]	105,163 children (8 to 18 years of age)	Longitudinal study	Children who had experienced at least one depressive event had a higher average BMI than children without such an experience; the older the children, the stronger the association.
Olive et al. (2017) [25]	791 healthy Australian children (7 to 8 years of age)	Longitudinal study	Both boys and girls with higher depressive symptoms had a higher percentage of body fat.
Nagata et al. (2018) [26]	14,322 U.S. adolescents	Longitudinal study	Depressive symptoms were associated with increased odds of unhealthy weight control behaviors in females; no association was found in boys.
Pine et al. (2019) [27]	231 preschoolers in St. Louis, MI, USA	Longitudinal study	There was a significant positive association between preschool depressive symptoms and adolescent BMI.
Moradi et al. (2020) [28]	NA	Meta-analysis	No association was observed between overweight and the risk of depression (pooled risk estimate: 1.00, 95% CI: 0.92–1.08, *p* = 0.97). Moreover, there was not any relationship between obesity and the risk of depression (pooled risk estimate: 1.08, 95% CI: 0.97–1.21, *p* = 0.17).

**Table 3:** Characteristics of studies that evaluated the association between childhood obesity and depression.

Author (Year)	Sample and Country	Study Type	Main Findings
Topçu et al. (2016) [29]	167 children with obesity and 200 normal-weight children aged 9–16 years	Case-control study	Obesity in children was associated with a significantly higher rate of depression and anxiety, and lower self-esteem scores. There were significant differences among them with obesity and control groups in terms of the total score of CDI [12 (4–39)] versus [8 (3–19)]; *p* < 0.001
Sepulveda et al. (2019) [30]	100 preadolescents (aged 8 to 12 years) from Madrid, Spain	Case-control study	Obese preadolescents reported higher levels of depression
Lindberg et al. (2020) [31]	12,507 Swedish children aged 6–17 in the Swedish childhood obesity treatment register (BORIS, 2005–2015) compared with a matched group of 60,063 children from the general population	Case-control study	Obesity was a significant risk factor for anxiety and depression. Girls with obesity had a 43% higher risk of anxiety and depression compared with girls in the general population. A similar result held for boys (adjusted HR, 1.33).
Sepulveda et al. (2018) [32]	170 children aged 8 to 12 years from different health centers in Madrid, Spain	Cross-sectional study	Five percent of the sample was diagnosed with major depression
Yang et al. (2018) [33]	197 elementary school students and 461 middle school students	Cross-sectional study	Childhood obesity can cause depression and reduce children’s quality of life because of their distorted body perception
Blanco et al. (2019) [34]	50 Spanish preadolescents with obesity	Cross-sectional study	Obese children reported higher depression.
Sepulveda et al. (2020) [35]	Nine Spanish families of children aged 8 to 12 years	Cross-sectional study	Positive association between weight status and depression (measured through CDI).
Lin et al. (2021) [36]	445 Taiwanese children aged 13 to 15 years	Cross-sectional study	No difference in depression between normal-weight and overweight/obese children (4.85 ± 3.53 vs. 4.81 ± 3.96)
Lim et al. (2016) [37]	759 high-grade elementary school students, 609 middle school students, and 496 high school students	Longitudinal study	Obesity was among the meaningful variables of depressive symptoms from childhood to adolescence.
Gibson et al. (2017) [38]	212 children aged 8 to 13 years	Longitudinal study	Overweight girls and girls with obesity reported greater impairment in depression as well as in other areas of psychological difficulties than normal-weight girls. No such association was found for boys.
Sanders et al. (2015) [39]	NA	Systematic review	A high depression prevalence in overweight children and children with obesity was identified in Australian children and adolescents
Quek et al. (2017) [40]	51,272 participants	Meta-analysis	There was a positive association between childhood and adolescent obesity and depression and more severe depressive symptoms in the groups with obesity.
Köhler et al. (2018) [41]	NA	Systematic review (umbrella review)	Obesity and metabolic abnormalities constituted risk factors for depression.
Sutaria et al. (2019) [42]	143,603 children	Meta-analysis	Obese female children had significantly increased odds of concurrent and future depression compared with non-obese female children.
Smith et al. (2020) [43]	NA	Literature review	Psychological conditions, such as low self-esteem, depression, and eating disorders can result from excess weight in childhood.

## Data Availability

Not applicable.
